# Celiac Disease Management in School Environments: An Interdisciplinary Narrative Review of Operational, Psychosocial, and Nutritional Barriers

**DOI:** 10.3390/nu18183042

**Published:** 2026-09-17

**Authors:** Alexandra Mpakosi, Vasileios Cholevas, Stamatios Cholevas, Argyro Pastrikou, Aikaterini Fragkiadaki, Foteini Tziraki, Ioannis Vogiatzis, Alexandra Lianou, Ioannis Tzouvelekis, Thomas Rallis, Maria Mironidou-Tzouveleki, Andreas G. Tsantes, Rozeta Sokou

**Affiliations:** 1 Department of Immunology, General Hospital of Nikaia “Agios Panteleimon”, 18454 Piraeus, Greece; argiropastr@gmail.com (A.P.); kathy_fragi@yahoo.gr (A.F.); paliotziraki@hotmail.com (F.T.); jvoyia.syr@gmail.com (I.V.); 2 School of Medicine, University of Bologna, 40126 Bologna, Italy; billcholevas34@gmail.com; 3 School of Pharmacy, European University of Cyprus, Diogenes 6, Engomi, 2404 Nicosia, Cyprus; stam17112004@gmail.com; 4 Neonatal Intensive Care Unit, General Hospital of Nikaia “Agios Panteleimon”, 18454 Piraeus, Greece; alexlianou95@gmail.com; 5 School of Agricultural Technology, Food Technology and Nutrition, Alexander Technological Educational Institute of Thessaloniki, 57400 Thessaloniki, Greece; tzouvelekisgiannis@yahoo.gr; 6 Thoracic Surgery Department, “Theageneio” Cancer Hospital, 54639 Thessaloniki, Greece; thrallis@gmail.com; 7 Department of Pharmacology, School of Medicine, Faculty of Health Sciences, Aristotle University of Thessaloniki, 54124 Thessaloniki, Greece; mmyronidauth@gmail.com; 8 Laboratory of Haematology and Blood Bank Unit, “Attikon” Hospital, School of Medicine, National and Kapodistrian University of Athens, 12462 Athens, Greece; agtsantes@med.uoa.gr; 9 Neonatal Department, Aretaieio Hospital, National and Kapodistrian University of Athens, 11528 Athens, Greece; rosesok@med.uoa.gr

**Keywords:** gluten-free diet, celiac disease, school kitchens, pediatric nutrition, cross-contact, school health policy, socio-economic disparities, peer stigmatization, surface sanitation

## Abstract

**Background**: Pediatric celiac disease (CD) prevalence is rising, estimated at 2% to 3% in select screening cohorts. Affected children face significant environmental, psychosocial, and nutritional barriers within institutional school settings. **Methods**: This narrative review synthesizes published clinical trials, cohort studies, and food science data to examine infrastructure vulnerabilities and care gaps within educational environments. **Results**: High-volume school kitchens present potential mechanical cross-contact risks via convection currents and shared equipment. Standard alcohol hand sanitizers demonstrate negligible mechanical or chemical efficacy against inert gliadin proteins, suggesting the need for surfactant-based washing protocols. In classrooms, tactile wheat-based materials introduce non-dietary ingestion pathways. These collective environmental challenges may contribute to social exclusion, peer stigmatization, and heightened risks for childhood mood variations. Furthermore, many commercial gluten-free alternatives lack routine fortification, potentially compounding celiac-related micronutrient deficiencies. **Conclusions**: Reactive, individualized accommodations may provide insufficient protection. Improving student safety and well-being supported by this narrative evidence points toward a general framework of structured kitchen guidelines, universal classroom adaptations, and jurisdiction-dependent administrative coordination.

## 1. Introduction

**Figure 1:**
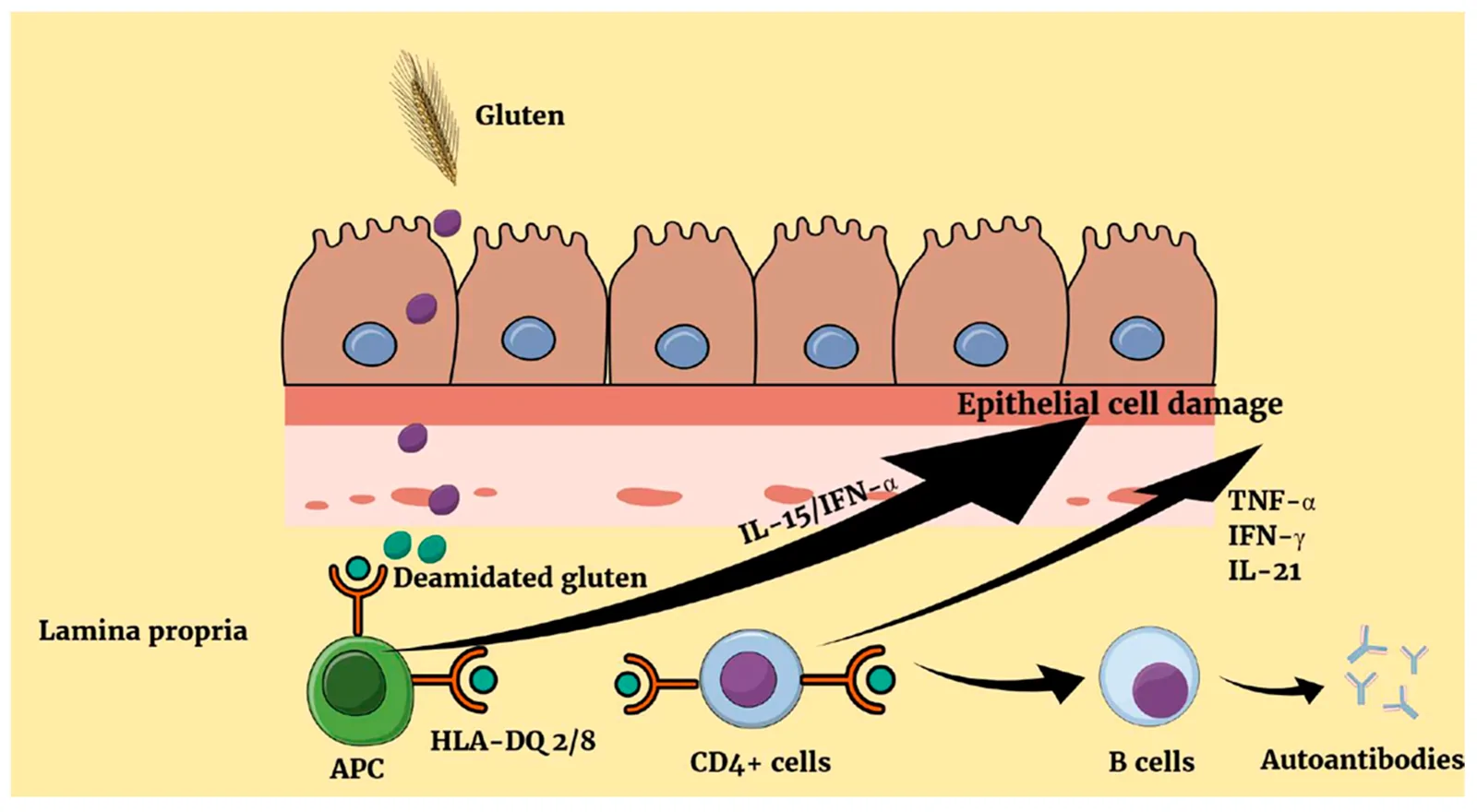
Pathophysiological Mechanism of Celiac Disease. Ingested gluten resists digestion, yielding resilient α-gliadin peptides that cross the epithelial barrier into the lamina propria. The enzyme tissue transglutaminase (tTG) deamidates these peptides, introducing negative charges that maximize their binding affinity to genetic HLA-DQ2/HLA-DQ8 receptors on antigen-presenting cells. This binding triggers helper CD4^+^ T cells, launching a robust, localized Th1-mediated cytokine response (IFN-γ, TNF-α) and signaling B cells to produce diagnostic anti-tTG and anti-EMA autoantibodies. Concurrently, stress-induced IL-15 expression by enterocytes activates cytotoxic intraepithelial lymphocytes (IELs). These activated killer cells directly destroy healthy enterocytes, flatlining the small bowel architecture into crypt hyperplasia and progressive villous atrophy, which drives systemic pediatric malabsorption. **Arrow pathways: Horizontal arrows:** Depict the downstream activation from activated CD4+ T cells to B cells, and the final differentiation into autoantibody-secreting plasma cells. **Thick upward arrow:** Illustrates the innate cytotoxic pathway driven by enterocyte stress signals (IL-15/IFN-α) leading to epithelial cell damage. **Thin upward arrow:** Illustrates the adaptive Th1-mediated cytokine response (TNF-α, IFN-γ, IL-21) driving chronic enterocyte destruction and villous atrophy. **Note: This figure is an original work designed and created by the author Alexandra Mpakosi using the Mind the Graph platform (mindthegraph.com).** tTG: tissue transglutaminase; APC: antigen-presenting cell; anti-EMA autoantibodies: anti-endomysial autoantibodies.

Celiac disease (CD) is a systemic autoimmune disorder triggered by the dietary ingestion of gluten grains (wheat, barley, and rye) in genetically predisposed individuals. Gluten proteins are rich in proline and glutamine, making them highly resistant to normal gastric and pancreatic enzyme breakdown. The undigested protein fragment, gliadin, breaches the intestinal epithelial barrier to enter the lamina propria. There, the endogenous enzyme tissue transglutaminase (tTG) deamidates the gliadin peptides. This introduces a negative electrical charge that vastly increases their immunogenicity [1,2]. These modified, negatively charged peptides fit precisely into the antigen-presenting binding grooves of specific human leukocyte antigen surface molecules. Specifically, HLA-DQ2 or HLA-DQ8 genotypes are present in nearly all affected individuals. However, it is worth noting that although approximately 40% of the general population carries these genes, only about 3% ultimately develop the disease, suggesting that—beyond gluten—other environmental factors are involved in the disease’s pathogenesis [3,4]. Antigen-presenting cells display these bound peptides to helper CD4 T cells. This triggers a massive, localized inflammatory cascade marked by the secretion of pro-inflammatory cytokines like interferon-gamma. Concurrently, intraepithelial lymphocytes are activated, transforming into killer cells that attack healthy enterocytes [1]. The condition causes a chronic, immune-mediated enteropathy affecting the small intestinal mucosa, leading to marked intraepithelial lymphocytosis, crypt hyperplasia, and progressive villous atrophy. The destruction of these villi—which are responsible for nutrient absorption—ultimately results in malabsorption syndromes (Figure 1) [4].

However, CD must be consistently distinguished from IgE-mediated food allergies. While both conditions necessitate strict ingredient containment, their underlying immunological mechanisms, temporal presentation of symptoms, and long-term clinical consequences differ fundamentally. IgE-mediated food allergies represent immediate hypersensitivity reactions capable of triggering acute, life-threatening anaphylaxis within minutes of exposure [5]. Conversely, CD is a chronic, cell-mediated autoimmune enteropathy driven by dietary gluten in genetically predisposed individuals. The resulting inflammatory cascade damages the small intestinal mucosa over time, leading to progressive villous atrophy and systemic malabsorption rather than acute anaphylactic collapse [4].

CD features both gastrointestinal and extraintestinal complications [6,7,8]. The global prevalence of CD is approximately 1% [9]. However, recent clinical screenings indicate an increasing prevalence in the pediatric population, reaching 2% to 3% among various groups of children and adolescents. Indeed, mass screening programs have confirmed these observed changes, with the prevalence in the pediatric population now hovering around 2% to 3% in countries such as Sweden [10,11,12].

The school environment is a primary source of inadvertent gluten exposure, physical stress, and social vulnerability for children with CD. Although schools are intended to be safe spaces for development, they pose significant risks, as daily school and social activities can directly threaten a child’s medical stability and emotional well-being. Untreated or undiagnosed CD has been shown to negatively impact the school performance of pediatric patients who may be absent from school or unable to concentrate or attend school [13]. Additionally, children and adolescents with silent or untreated CD may be associated with an increased incidence of behavioral disorders and the occurrence of depression [14]. While the clinical management of celiac disease is well-established in ambulatory settings, addressing its management within institutional school environments has emerged as an urgent public health imperative. Globally, children spend a significant portion of their developmental years within educational facilities, where unstructured meal periods, shared infrastructure, and standardized curricula frequently intersect to create unmanaged gluten exposure pathways. Because trace gluten ingestion drives progressive enteropathy and chronic systemic deficiencies even in asymptomatic cohorts, sub-optimal institutional containment represents a widespread threat to pediatric growth, cognitive development, and long-term academic attainment. Furthermore, the lack of standardized, equitable accommodation frameworks introduces profound socioeconomic disparities, leaving low-income and food-insecure families disproportionately vulnerable to poor disease management and subsequent healthcare utilization.

The primary objective of this narrative review is to provide a comprehensive, interdisciplinary synthesis of the environmental, psycho-sociological, socio-economic, and nutritional barriers that children with CD encounter within the school setting. We hypothesize that a reliance on reactive, individualized accommodations fails to maintain cumulative gluten exposures below clinically reactive thresholds, and that mitigating these multi-systemic risks requires a transition toward universal classroom designs and standardized kitchen protocols. By evaluating these intersecting domains, this study aims to identify systemic operational gaps and propose an evidence-based, flexible framework to optimize institutional safety, promote social equity, and improve clinical outcomes for celiac youth.

## 2. Materials and Methods

### 2.1. Search Strategy

A comprehensive literature search was conducted across PubMed, MEDLINE, and Scopus databases to identify relevant peer-reviewed publications exploring CD management within educational environments. The final literature search was executed on 26 July 2026, capturing publications indexed between January 2000 and July 2026. Language restrictions were applied, limiting the final inclusion to articles published exclusively in the English language.

The search strategies relied on combinations of MeSH terms and text keywords, structured around three core areas:

1. Environmental risks: (“celiac disease” OR “coeliac disease” OR “gluten”) AND (“school” OR “cafeteria” OR “institutional kitchen” OR “cross-contact” OR “surface sanitation” OR “hand sanitizer”).
2. Psychosocial and economic barriers: (“celiac disease” OR “coeliac disease”) AND (“pediatric” OR “adolescent” OR “student”) AND (“peer stigmatization” OR “bullying” OR “food insecurity” OR “socioeconomic”).
3. Nutritional parameters: (“gluten-free diet” OR “celiac disease”) AND (“school meal” OR “school lunch” OR “nutritional deficiency” OR “fortification” OR “pseudocereals” OR “whole foods”).

### 2.2. Inclusion and Exclusion Criteria

Studies were eligible for inclusion if they fulfilled the following criteria: (1) original peer-reviewed research, clinical trials, prospective/retrospective epidemiological cohorts, food science evaluations, or institutional public health manuals; (2) focus on pediatric or adolescent populations aged 0–18 years; and (3) direct assessment of environmental, nutritional, psycho-social, or financial variables affecting children with CD inside primary or secondary school environments.

Publications were strictly excluded if they consisted of non-peer-reviewed literature, consumer lifestyle blogs, editorials, conference abstracts lacking complete data panels, or self-published dietary manuals.

### 2.3. Study Selection and Data Extraction Protocol

To ensure transparency and maximize reproducibility, a rigorous three-stage screening protocol was established. In the first stage, duplicates were mechanically removed. In the second stage, two review authors (A.M. and R.S.) independently evaluated titles and abstracts against the predetermined eligibility criteria. In the third stage, full-text articles of all potentially relevant studies were retrieved and reviewed in duplicate. Data extraction was performed independently by the same investigators (A.M. and R.S.). Extracted data parameters included primary author, year of publication, geographic cohort location, study design, exact operational vectors examined and key clinical or analytical outcomes. Any operational discrepancies or disagreements regarding study eligibility or data synthesis during the screening and extraction phases were resolved through open consensus, with a third investigator (V.C.) serving as an independent arbitrator when necessary.

### 2.4. Data Availability and Ethical Approval

As a narrative review analyzing secondary published literature, this study did not generate primary data or large datasets. For the same reason, this work did not involve direct human or animal interventions, requiring no institutional review board (IRB) ethical approval or corresponding approval codes. All source documents and protocols utilized are fully disclosed and accessible through the included bibliography.

## 3. Environmental Risks and Cross-Contact in Schools

### 3.1. The School Cafeteria

#### 3.1.1. The Institutional Sourcing Paradigm and Labeling Complexities

The institutional school cafeteria represents a premier high-risk vector for accidental gluten cross-contact due to the inherent conflict between high-volume food production, shared infrastructure, and a lack of specialized staff training. Bioletti et al. conducted a comprehensive retrospective analysis evaluating the administrative and operational management of gluten-free meals across primary and secondary school catering services in the Piedmont region [15]. Their findings revealed a striking systemic deficit: only 29% of the audited educational catering facilities demonstrated adequate execution across all evaluated parameters—including procurement controls, storage, process analysis, equipment control, packaging, transportation, meal distribution, self-monitoring safety plans, and quality assessment. Conversely, a profound 71% of school food services were deemed inadequate in at least one of these critical operational domains, highlighting a pervasive baseline failure in institutional celiac containment protocols [15].

Given that the multi-systemic pathognomonic cascade of CD is triggered by trace ingestions of gluten grains, all foods derived from or cross-contaminated by wheat, rye, barley, and non-certified oats must be systematically eliminated from pediatric school diets [16]. Consequently, menu formulation must strictly exclude processed culinary staples containing hidden wheat derivatives, such as traditional soy sauces, concentrated meat broths, commercial soups, and breakfast cereals utilizing barley malt or malt-derived flavorings [17].

Furthermore, invisible fractions of gluten are frequently integrated into processed charcuterie matrices (such as commercial hot dog fillings), used as binders in texturized meat products, or introduced via standard processing brines injected into commercial poultry [18]. Sourcing protocols must dictate that all food products procured by school nutrition authorities carry a certified “gluten-free” label that is actively audited prior to service [17].

While global regulatory bodies—including the Codex Alimentarius, the European Commission, and the Food and Drug Administration (FDA)—mandate a strict analytical limit of less than 20 mg/kg for gluten-free designations, labeling compliance alone does not guarantee absolute safety [17]. Complex, multi-ingredient industrial food supplies frequently mask “hidden gluten” under ambiguous ingredient declarations like modified starch, texturized plant protein, or natural spice carriers [19,20,21].

Moreover, naturally gluten-free grains (such as raw sorghum, millet, or buckwheat) that bypass certified gluten-free processing channels present an unmanaged cross-contamination risk due to shared agricultural harvesting machinery and transport infrastructure [22]. Consequently, institutional kitchens cannot rely solely on the intrinsic, raw classification of an ingredient; they must exclusively source products that maintain a verified, sub-20 ppm (20 parts per million) analytical seal [17]. However, it is critical to distinguish regulatory benchmarks from situational clinical thresholds. While global regulatory frameworks mandate an analytical limit of less than 20 mg/kg (20 ppm) for individual food labeling compliance, this value represents a commercial baseline rather than a universal clinical threshold applicable to single exposure events or environmental surfaces [23,24]. Clinical manifestations depend on the cumulative daily mass of gluten ingested (typically quantified as a threshold of 10–50 mg per day for sensitive individuals) rather than isolated contamination parts-per-million concentrations [23,25]. Consequently, analytical surface contamination metrics cannot be directly extrapolated to definitive clinical pathology without accounting for the frequency, mass, and direct ingestion pathways of the specific contact vector [24].

#### 3.1.2. Shared Cooking Surfaces and Equipment

When safe ingredients are manipulated within the educational environment, preventing physical contact with gluten-containing substrates is a strict medical necessity. In high-volume institutional food lines, cross-contamination vectors manifest rapidly across shared utensils and prep zones [26]. Empirical data from Studerus et al. demonstrates that significant gluten transfer occurs dynamically within commercial food service settings through simple mechanical vectors, specifically proving that the utilization of shared serving ladles, spoons, and tongs during rapid food assembly is sufficient to introduce toxic protein fractions into safe meal portions [27]. Managing CD in this setting requires strict mitigation strategies to minimize cumulative trace exposure [28]. However, standard school kitchen workflows are inherently built for speed and mass output, which directly compromises safety [28].

Indeed, industrial kitchen equipment is designed for multi-use efficiency, creating structural vectors for cross-contact that are difficult to eliminate without dedicated infrastructure [29]. For example, forced-air convection ovens use powerful internal fans to circulate high-velocity heat. When gluten-containing products (such as standard pizza or breaded chicken pieces) are baked uncovered next to gluten-free products, forced air circulation carries microscopic gluten particles and airborne flour dust directly onto safe foods [30].

Furthermore, shared commercial toasters, griddles, and flat-top grills act as critical hotspots for the dense accumulation of wheat crumbs and grease residues. Standard aesthetic wiping with a damp towel fails to break down or denature the highly stable gluten protein matrix, leaving behind invisible yet highly reactive residues [27].

At the thermal level, gluten proteins are completely heat-stable and are not denatured or destroyed at standard frying temperatures. When breaded items are processed in a commercial fryer, gliadin fractions slough off and remain actively suspended in the liquid medium, introducing measurable quantities of gliadin into any naturally gluten-free item subsequently submerged in the same shared oil [31].

Beyond the immediate kitchen boundary, this risk extends into unstructured student environments. Cafeteria tables, classroom desks, and communal microwave ovens are rarely subjected to the rigorous, friction-based decontamination required to strip the sticky protein film from hard surfaces. This janitorial gap makes accidental surface-to-hand and surface-to-food cross-contact highly likely during unstructured lunch and snack periods, creating a continuous hazard path for celiac youth [32].

### 3.2. Classroom and Art Room Contaminants

Schools are filled with hidden sources of gluten that extend far beyond the cafeteria tray. Classrooms and art rooms pose significant risks of gluten exposure as everyday educational materials can lead to accidental ingestion via hand-to-mouth contact. Especially, preschool and early childhood classrooms rely heavily on tactile stimulation, creating direct physical vectors for cross-contact with gluten, leading to subsequent hand-to-mouth contamination during snack time [33]. A recent study examined the environmental risk vectors across early education settings from a clinical management perspective. It documented how every day, non-cafeteria activities (including art class projects, holiday craft-making, and unstructured recess/sensory play) may serve as primary drivers for accidental exposure due to a lack of environmental containment [34]. Indeed, several standardized commercial modeling compounds, including modeling clay/dough (for example Play-Doh), are prepared directly from wheat flour [33]. Additionally, papier-mâché art (with newspaper and adhesive made from wheat flour, salt, and water), dry or cooked and dyed pasta are all used at this age and constitute hidden sources of gluten for children with CD [33]. Shared plastic toys may also become contaminated with microscopic traces of gluten, which can easily be transferred between students. Weisbrod et al. attempted to measure the transfer of gluten from school supplies to gluten-free foods that a child with CD might eat [33]. Thirty students aged 2 to 18 participated in five experiments that included activities such as Play-Doh, baking, papier-mâché, dry and cooked pasta on a sensory table. After the activities, gluten levels were measured on separate slices of gluten-free bread that were rubbed on the participants’ hands and table surfaces. They found that the use of papier-mâché, cooked pasta in sensory panels and baking programs resulted in gluten transfer well above the 20 ppm limit set by the Codex Alimentarius Commission. On the other hand, Play-Doh and dry pasta resulted in a small transfer of gluten to gluten-free bread. Soap and water proved to be the most effective washing method for removing gluten [33].

In higher grade levels, such as middle and high school, biological materials containing wheat and gluten are often used to demonstrate scientific principles—for example, in biology experiments and DNA extraction labs, fermentation and dough experiments, and studies involving bioplastics and green chemistry [31,35].

## 4. Inadequate Staff Training

School food service staff experience high turnover rates and lack standardized training regarding systemic autoimmune diseases. For instance, staff members often confuse celiac disease with a lifestyle choice or a mild wheat intolerance. This dangerous misconception leads to a lack of urgency and inadequate management of gluten-free meals. Untrained staff might assume that removing a standard wheat bun from a burger or a crouton from a salad makes the meal safe [36,37,38]. However, for a person with CD, the microscopic traces remaining after brief contact are enough to trigger a harmful autoimmune reaction [30,39]. Using the same utensils and serving spoons for different foods also directly puts children with CD at risk. Furthermore, without specialized training in systematically reading labels, staff often overlook hidden ingredients—such as hidden wheat—in the pre-packaged and highly processed products they serve to children [40].

However, the vulnerabilities introduced by institutional school food services extend far beyond baseline deficiencies in staff disease literacy. The structural architecture of modern public school lunch infrastructure is fundamentally optimized for high-volume throughput and maximum service velocity, features that run directly counter to the absolute zero-tolerance precision required for celiac safety. During peak lunch service rushes, compressed time windows force food service workers into states of severe cognitive tunneling. In these high-stress environments, frontline staff focus exclusively on speed and quantity, deprioritizing complex, multi-step safety tasks [28].

Furthermore, this operational deficit is compounded by a widespread institutional misconception regarding “visual cleanliness” versus molecular trace protein contamination [41]. Because standard culinary sanitation codes emphasize biological decontamination—utilizing heat or chemical sanitizers to destroy living bacterial pathognomonic clusters—staff lack the molecular training to understand that gluten is an inert, highly stable protein matrix. Food preparation workers consistently operate under the false assumption that a flat-top grill, shared convection tray, or serving ladle is entirely safe if it appears clean to the naked eye [41]. They fail to recognize that an invisible residue or a single microscopic crumb measuring less than 20 ppm is biologically sufficient to trigger an autoimmune cascade and induce progressive enteropathy in a developing child’s duodenal tissue, regardless of whether the exposure elicits immediate, overt clinical symptoms.

These internal kitchen errors are exacerbated by structural blind spots within institutional supply chains and shift-to-shift communication loops. Public school kitchens navigate frequent supply chain variances, resulting in regular, unannounced product or bulk brand substitutions from regional distributors [42]. While a kitchen manager may audit an ingredient panel on a Monday, frontline serving personnel rarely re-verify micro-ingredient configurations or trace statements on modified mid-week delivery shipments, allowing hidden wheat stabilizers or malt flavorings to bypass containment streams [43]. This risk is heightened by high staff turnover, which creates a communication void between morning preparation shifts and afternoon service windows [43]. Finally, this system operates without independent accountability; standard municipal health inspections focus almost exclusively on pest control and temperature thresholds, completely lacking rigorous third-party audits of allergen critical control points (CCPs) [44]. Without external regulatory enforcement, celiac containment protocols inside the school cafeteria degrade into voluntary, poorly managed guidelines rather than strictly enforced medical interventions [44].

## 5. Psycho-Sociological Parameters of Pediatric Celiac Disease Inside the School Environment

Sharing food is a fundamental bonding ritual in child development. However, these everyday social interactions become primary drivers of isolation and peer stigmatization for children with CD [34]. Systematic exclusion from such moments imposes a heavy psychological burden, as it isolates these children from their peers and renders them particularly vulnerable to bullying related to their health condition, as well as to long-term emotional distress. The very protocols designed to ensure a child’s physical safety often cause serious emotional and social harm [45]. For example, forcing a child to sit at a gluten-free table creates a form of institutionalized exclusion. Sitting apart makes a child’s medical condition highly visible to the entire student body. This constant public labeling can cause deep self-consciousness and embarrassment, turning a routine lunch break into a source of daily anxiety [46,47]. While children of the same age sit together, share snacks, and form friendship groups, the child facing segregation remains on the social sidelines. This dynamic hinders his ability to develop natural, everyday relationships. Exclusion from classroom cooking activities—often imposed by teachers who believe they are protecting the child—deprives them of valuable shared learning experiences. In addition, gluten is central to school celebrations and birthday parties. Substitutes for children with CD are often forgotten, inadequate, or cross-contaminated. This situation reinforces a sense of being an “outsider,” while the need to explain why they cannot participate gives rise to deep disappointment. Managing a chronic illness during critical developmental years forces children to view themselves as “sick” or “different” [47]. This self-perception can hinder the development of self-esteem and peer relationships. Over time, children may internalize this experience, feeling like a burden to their class or peer group [48]. A massive nationwide matched cohort tracking study has revealed that children diagnosed with CD present a 1.4-fold greater risk of developing future psychiatric disorders [47]. It highlights a definitive inflation in mood disorders, anxiety disorders, behavioral disruptions, and emotional distress stemming directly from the combined biological and psychological realities of navigating the chronic condition among peers [47].

In many instances, children are frequently teased by peers who view them as simply “picky eaters,” “over-the-top,” or “fussy.” In extreme cases, children with CD may face bullying, including verbal taunting and dangerous physical acts. To stop the teasing and to fit in with their peers, developing children face intense psychological pressure to hide their illness. This desire for social acceptance can lead to dangerous behavioral changes, with children secretly consuming gluten-containing foods—simply to avoid standing out negatively among friends—thereby risking serious internal damage [49,50].

## 6. Socio-Economic Inequalities in School-Based Management

The strict medical management of pediatric CD in the school environment acts as a significant factor in exacerbating socioeconomic inequalities. Given that a gluten-free diet is a mandatory medical requirement rather than a lifestyle choice, the school system’s failure to provide safe, subsidized options places a disproportionate financial and practical burden on low-income families [38]. This situation leaves vulnerable children exposed to both food insecurity and health risks [46]. Families from lower socioeconomic backgrounds face severe financial pressure in their efforts to secure safe food options within the public school system. A recent study revealed that over 50% of food-insecure families never use free school breakfast or lunch programs due to well-founded parental anxieties regarding unsafe kitchen facilities and the school’s structural inability to prevent cross-contamination during high-volume service rushes [51]. Furthermore, a multi-variable clinical cohort study established that decreased health literacy, lower neighborhood income, and institutional social adversities are mathematically linked to poor adherence and elevated antibody indices. The study also connected the lack of parental advocacy power and a school’s insufficient healthcare staffing directly to poor physical disease markers in pediatric cohorts [49]. Low-income families who qualify for state-subsidized free school breakfast and lunch programs are often unable to benefit from them, as schools cannot guarantee meals that are safe and free from the risk of cross-contamination. This shifts the entire financial burden of daily meal preparation onto the family budget. Purchasing certified gluten-free staple foods (such as bread, flour, and snacks) entails a substantiated price increase of approximately 150% to 500% compared to conventional wheat-based products [52,53,54]. For a family living below the poverty line, this steep rise in costs can lead to food insecurity, forcing them to make difficult choices between purchasing safe food and paying utility bills [55,56]. Affordable, long-shelf-life gluten-free substitutes are often highly processed products that are high in calories and low in fiber, iron, and B vitamins. Low-income students who lack access to fresh, nutritious gluten-free options are at increased risk of nutritional deficiencies, which can hinder their growth and development [57,58].

The structural management of pediatric CD within educational systems is heavily dependent on regional legal and administrative infrastructure. Consequently, the institutional recommendations detailed herein constitute a general operational framework rather than universally applicable legal mandates. Requirements regarding the necessity of specialist diagnostic documentation, national health insurance coverage, state-subsidized school meal programs, and campus-specific nursing allocations vary substantially across countries. While centralized models provide valuable reference parameters, their local feasibility remains contingent upon national legislative frameworks, municipal school funding mechanisms, and regional socioeconomic resource distribution. For example, a school’s ability to safely accommodate a child with CD is often directly linked to funding levels. Schools with limited financial resources often lack dedicated food preparation areas or separate appliances, relying instead on mass-produced, pre-packaged meals that are simply heated and served [38]. Schools in high-poverty areas often face chronic understaffing and high staff turnover, making it extremely difficult to both oversee kitchen operations and maintain ongoing training on cross-contact prevention protocols. Furthermore, schools with insufficient funding lack specialized staff or often share a single nurse across multiple school units, leaving students without immediate medical supervision in the event of accidental gluten exposure. Sahın et al. revealed the critical presence of the school nurse as a primary factor in successful student management plans [34]. This validates the vulnerability of children in low-income or understaffed school districts that lack full-time, campus-dedicated health personnel to handle unexpected exposures [34]. Weisbrod et al. studied celiac accommodations across various educational units. They revealed wide-ranging inconsistencies and inequalities in how schools offer gluten-free selections, kitchen safeguards, and staff training. The data supported the premise that lower-resource public schools lack standardized allergen-safe options, frequently relying on un-segregated, pre-packaged heat-and-serve loops that present high cross-contact risks [38]. In contrast, well-funded schools are more likely to employ full-time school nurses and specialized nutritionists capable of developing and implementing personalized nutrition plans [38]. To qualify for official accommodations within the school environment, families must submit official diagnostic documentation from a pediatric gastroenterologist. For families who are uninsured or have inadequate insurance coverage, the cost of specialist consultations, diagnostic blood tests, and endoscopies poses a significant barrier. Roy et al. proved that families of lower socioeconomic status face vast disparities in diagnostic access and specialty health-seeking capabilities [59]. Their study revealed how financial barriers—specifically out-of-pocket costs for specialist copays, diagnostic blood counts, and mandatory endoscopic duodenal biopsies—delay diagnosis. This acts as a barrier preventing low-income children from securing the official paperwork required for legal school meal modifications and accommodation plans (Table 1) [59].

Furthermore, an analysis of the available literature reveals a profound geographical imbalance in global public health surveillance. Systematic, empirical data evaluating direct molecular surface testing remains heavily concentrated in high-income Western regions, specifically North America and parts of Western Europe [18,26,29].

Conversely, severe epidemiological data gaps persist across low- and middle-income countries, where mass institutional school feeding systems operate under limited infrastructure budget conditions. In these high-poverty regions, surveillance frameworks are practically non-existent, leaving public health authorities without reliable containment baselines. Bridging these global surveillance gaps is essential to designing scalable, localized health policies that protect celiac youth across differing geopolitical landscapes (Table 2).

## 7. The Nutritional Deficit of Commercial GFDs

### 7.1. Ultra-Processed Food Matrices and the NOVA Classification

The nutritional quality of a strict GFD is frequently compromised by a heavy reliance on commercial substitutes [62]. Under the NOVA food classification framework, which categorizes agricultural products based on the extent and purpose of industrial processing, the vast majority of commercially engineered gluten-free alternatives fall directly into Group 4: Ultra-Processed Foods (UPFs) [63]. As detailed by Melini and Melini, when industrial manufacturers remove gluten from grain matrices, they must substitute the structural, cohesive, and viscoelastic properties typically provided by wheat proteins [16]. This modification requires the systematic formulation of complex, industrial mixtures, replacing whole-grain flour with highly refined, low-protein starches, including refined rice starch, corn starch, potato starch, and tapioca flour [64,65]. Because these isolated starches inherently lack structural elasticity and palatability, the formulation of these Group 4 UPFs necessitates the intensive addition of cosmetic emulsifiers, hydrocolloids, hydrogenated fats, simple sugars, and high-fructose corn syrups to mimic standard culinary textures [17,66].

Recent longitudinal evidence from Nestares et al. reinforces these findings, demonstrating that UPFs constitute the primary daily energy source for pediatric celiac cohorts, accounting for over 57% of total caloric intake [67]. This intake is heavily concentrated around specific school-related eating periods, peaking dramatically during breakfast (~74%) and afternoon snacks (~81%). Crucially, Nestares et al. established that global UPF consumption patterns and their associated nutritional imbalances remain remarkably stable even after 12 months on a strict GFD. While patients over a 12-month period exhibit micro-dietary shifts—such as reducing their intake of basic processed foods (NOVA Group 3) and culinary ingredients (NOVA Group 2)—their systemic reliance on ultra-processed Group 4 alternatives remains deeply ingrained and structurally identical to healthy peer controls [67].

### 7.2. Metabolic Cascades and Micronutrient Deficiencies

The persistent consumption of these starch-heavy, ultra-processed gluten-free matrices introduces severe metabolic and clinical challenges for growing children:

- Glycemic Volatility: These ultra-processed substitute products feature an elevated glycemic index. For pediatric populations, consumption of these rapidly digestible starches induces sharp postprandial glucose excursions followed by reactive, insulin-driven hypoglycemia. This metabolic “rollercoaster” contributes to daytime cognitive fatigue, impaired classroom concentration, and long-term risks of childhood obesity and insulin resistance [17].
- Prebiotic Deprivation: Traditional whole grains possess an outer bran and germ layer rich in dietary fiber. In contrast, the starch substitutes used in Group 4 gluten-free formulations are largely stripped of fiber during industrial refining. This chronic dietary fiber deficit deprives the pediatric gut microbiome of vital prebiotic substrates, which alters microbial diversity and leads to sluggish bowel motility, abdominal discomfort, or functional constipation [68,69,70]. These symptoms can easily be confused with active gluten exposure, causing unnecessary anxiety for families and school health staff [71].
- The Fortification Deficit: In many jurisdictions, public health mandates require the artificial fortification of mass-produced wheat flours with essential trace minerals and B-complex vitamins to mitigate population-wide deficiencies [72,73]. However, these mandatory fortification frameworks rarely apply to gluten-free industrial starches [74]. Consequently, children relying heavily on ultra-processed gluten-free foods face a high risk of micronutrient deficiencies. This systematic lack of food fortification exacerbates the impairment of micronutrient absorption already caused by intestinal villous atrophy associated with CD. This dual vulnerability places pediatric patients at an extremely high risk of developing iron-deficiency anemia, delayed bone development, and suboptimal physical growth markers [67,75,76].

### 7.3. Practical Guidelines for Shifting from Processed Gluten-Free Snacks to Nutrient-Dense, Naturally Gluten-Free Whole Foods in School Meals

Switching from highly processed gluten-free alternatives to whole foods—which are nutrient-rich and naturally gluten-free—for a child’s school lunch is a crucial step toward reversing chronic micro-anemias and metabolic imbalances [77]. Typical school meals often rely on commercially available sliced white bread or gluten-free wraps. These products consist of highly processed starches that lack fiber and cause a sharp postprandial rise in glucose levels [78]. To optimize macro-nutritional intake, highly processed starches should be replaced with a structured foundation of minimally processed, naturally gluten-free carbohydrates. This selection includes sprouted ancient pseudocereals (such as quinoa and amaranth) as well as naturally gluten-free whole grains (such as millet and wild rice). These complex, low-glycemic-index options retain their germ and bran layers intact, providing B-complex vitamins and dietary fibers that contribute to sustained cognitive concentration during afternoon school activities [79]. Combining these complex, low-glycemic-index carbohydrates directly with a source of highly bioavailable iron—such as finely chopped roasted chicken breast, lean roast beef, or boiled eggs—is also considered a step in the right direction. To enhance the absorption of non-heme iron by the damaged enterocytes, adding an ascorbic acid (vitamin C) catalyst—such as raw bell pepper strips, strawberry slices, or cherry tomatoes—to the same section of the food container would be beneficial [80,81]. Instead of high-fat commercial snacks like chips, oven-roasted chickpeas (with olive oil), raw pumpkin seeds, or sunflower seeds are recommended [69]. These alternatives are naturally gluten-free and exceptionally rich in zinc, magnesium, and highly bioavailable essential fatty acids [69]. Incorporating raw plant fibers that retain their natural skin—such as unpeeled cucumber slices—into the diet provides valuable prebiotic substrates for reversing functional gut imbalances and sluggish motility, conditions often caused by the low fiber content characteristic of commercial diets for CD [17].

Secondary lactose intolerance is common in patients with CD due to damage to the mucosal brush border [69]. However, plant-based beverages should not be viewed as universally nutritionally equivalent to mammalian dairy. When evaluating dairy alternatives for growing children, the pediatric dietitian must carefully consider protein content, micronutrient bioavailability, age, and the child’s overall dietary pattern. Standard commercial plant beverages (such as almond or rice milk) are often significantly lower in protein than cow’s milk, which can compromise age-specific skeletal growth and daily macro-nutrient requirements during critical pediatric developmental phases. Consequently, if a dairy-free approach is clinically indicated, the selection must prioritize unsweetened plant-based beverages explicitly fortified with calcium and vitamin D3, within a broader diet that provides adequate structural protein from alternative sources [69]. However, for children who tolerate dairy, plain kefir (without added sugar) or Greek yogurt mixed with fresh blackberries or blueberries is recommended [82]. This combination provides a high concentration of bioavailable calcium and live active cultures, supporting the restoration of gut microbiome diversity [17].

To mitigate chronic micro-anemias, metabolic fluctuations, and macro-nutritional deficits, structural meal planning should focus on nutrient-dense, naturally gluten-free whole foods. However, because celiac enteropathy complicates micronutrient absorption in highly variable ways, dietary planning for children with CD must be strictly individualized and supervised by a registered pediatric dietitian [83].

Professional intervention is particularly critical when introducing raw fibers, nuts, seeds, and intensive iron-absorption strategies to support age-specific pediatric growth spurts without causing gastrointestinal distress. Furthermore, while the substitution of ultra-processed alternatives with whole foods is beneficial, specialized clinical monitoring is required to avoid localized functional imbalances (Table 3) [83].

## 8. Policy Recommendations

Implementing effective CD management in schools requires a shift from reactive, individualized adjustments to a proactive, systemic framework. Actionable policy proposals are needed, designed to integrate the health and education sectors. Such interventions ensure legal and operational safeguards for the child’s medical safety, financial stability, and socio-psychological well-being.

### 8.1. The Clinical–Educational Bridge

Educational institutions must remove communication barriers between clinical medicine and the school community by establishing a comprehensive, child-centered care team. A formal data-sharing process is required, through which the pediatric gastroenterologist provides clear diagnostic criteria and personalized dietary parameters directly to the school health team [84]. This structural link ensures that school actions are guided by medical oversight. Furthermore, the role of central compliance coordinator should be assigned to a specifically designated school nurse based at the school unit [85]. This healthcare professional should be responsible for incorporating medical guidelines, managing protocols for handling exposure incidents, and acting as a liaison between parents and teaching staff [86]. However, implementing a cohesive clinical–educational bridge is subject to significant cross-border variance in public policy and school health resources. Furthermore, the role of a campus compliance coordinator is constrained by national nursing allocation models; many public-school districts globally operate under a shared-nursing paradigm, where a single professional manages multiple academic facilities.

### 8.2. The Food Service and Counseling Loop

School nutrition directors should collaborate directly with school psychologists and guidance counselors to bridge the gap between physical health and mental well-being. While cafeteria managers oversee the physical safety and micro-structural containment of institutional food preparation, counseling personnel must proactively monitor affected students for clinical indicators of generalized anxiety, social withdrawal, or medical bullying driven by their chronic illness [34].

### 8.3. Mandatory Staff Education

Educational boards should implement mandatory, annual, and standardized training modules for all school personnel, including instructional faculty, administrative staff, and janitorial teams. This curriculum must explicitly define CD as a chronic, systemic autoimmune disorder, aggressively addressing and correcting the dangerous societal misconception that a gluten-free diet is merely a “lifestyle fad” or a behavioral preference [87].

### 8.4. HACCP Alignment in the School Kitchen

Institutional kitchens must deploy a specialized Hazard Analysis and Critical Control Points (HACCP) system engineered specifically for food allergen and gluten containment. This protocol mandates the structural implementation of dedicated preparation zones for gluten-free products, color-coded utensils to prevent tool cross-contact, protective covers or fan-disengagement standards for convection systems, and entirely separate, isolated circuits for commercial frying oil [88]. However, where institutional kitchen and classroom recommendations are extrapolated from established food-allergy literature, these adjustments must be tailored to celiac-specific criteria. For instance, while specialized HACCP protocols and allergen-safe zones are frequently adapted from pediatric food-allergy frameworks, their execution in the context of CD must focus on a cumulative parts-per-million threshold rather than acute systemic reactivity.

### 8.5. Universal Classroom Design

School boards must revise standardized academic curricula to eliminate non-ingestion-related gluten risks across all grade levels. Operational policy updates must ban wheat-flour modeling clays, raw-pasta sensory bins, and wheat-germ biological experiments in STEM laboratories. These hazardous materials should be replaced with naturally gluten-free alternatives for the entire student body, ensuring universal classroom design so that no child faces medical exclusion or public labeling [33].

### 8.6. Addressing Food Insecurity and Socio-Economic Gaps

The expansion of public funding streams for subsidized gluten-free meals or centralized bulk procurement of pseudocereals must be customized to align with local macroeconomic structures and public health legislation. To eliminate the regressive “gluten tax” that disproportionately burdens lower-income families, state public policies must guarantee universal, equitable access to safe nutrition.

#### 8.6.1. Subsidized Gluten-Free Meal Programs

Subsidized gluten-free meal programs must be structurally integrated into public infrastructure. Public funding streams should expand to absorb the cost-premium associated with certified gluten-free ingredients within the framework of universal free school breakfast and lunch systems [51]. School meal authorities must receive dedicated, ring-fenced funds for the procurement of verified gluten-free foods, ensuring that low-income, food-insecure students are not medically forced to opt out of free institutional meal loops [49].

#### 8.6.2. Bulk Procurement in the Supply Chain

State educational authorities should utilize centralized purchasing networks to execute the bulk procurement of naturally gluten-free agricultural staple foods, such as quinoa, brown rice, and legume-based flours. This supply chain strategy leverages institutional buying power to reduce per-unit costs, ensuring that school districts with limited funding maintain continuous access to safe, high-quality, and nutrient-dense ingredients [17].

#### 8.6.3. Eliminating Diagnostic Barriers

Public health policies must expand insurance coverage to fully include specialized diagnostic serological screening panels and endoscopic duodenal biopsies for uninsured or underinsured children. Providing free access to diagnostics ensures that families can obtain the official documentation required to qualify for legal accommodations [49].

### 8.7. Hygiene, Sanitation, and Symptomatic Management

Gluten-free items must be prepared in dedicated clean zones, processed using separate utensils, and handled by staff wearing clean, non-porous gloves [30]. While students may provide their own specialized lunchboxes, school nutrition programs must provide safe, equivalent gluten-free alternatives—such as fortified gluten-free breads, cereals, or pasta—during institutional meals to support a balanced nutrient profile. This accommodation fosters essential psychological normalization among peers, preventing social isolation and reducing the visibility of the student’s chronic illness [46].

Standard institutional cleaning protocols consistently fail because they rely on alcohol-based hand sanitizers, which possess negligible mechanical or chemical capability to dissolve or lift inert gluten proteins. Unlike living microorganisms whose cell membranes are disrupted by alcohol, gluten is an exceptionally stable, non-living protein matrix that requires physical, friction-based removal and fluid rinsing [33,89]. Relying on sanitizers simply dilutes and spreads the gliadin peptides more evenly across the epidermis, creating an invisible film that is easily transferred to downstream objects. Consequently, soap-and-water protocols utilizing active scrubbing must be strictly enforced immediately following tactile crafts, recess, or sensory bin usage, and always prior to eating [33].

Because inadvertent trace ingestion of gluten triggers a cell-mediated autoimmune response, affected students may develop subacute gastrointestinal manifestations, including abdominal pain, bloating, and diarrhea. Unlike IgE-mediated hypersensitivities, these gastrointestinal cascades typically do not manifest instantaneously. Instead, clinical or subclinical enteropathy develops progressively, with symptomatic onset often delayed by several hours or days following exposure. School health staff must implement a reliable communication channel to notify parents if a confirmed exposure occurs or if secondary symptoms manifest, including headaches, fatigue, or—particularly in adolescent cohorts—atypical neuropsychiatric features and sudden declines in concentration [90,91,92,93].

#### Effective Surface Protocols

**Figure 2:**
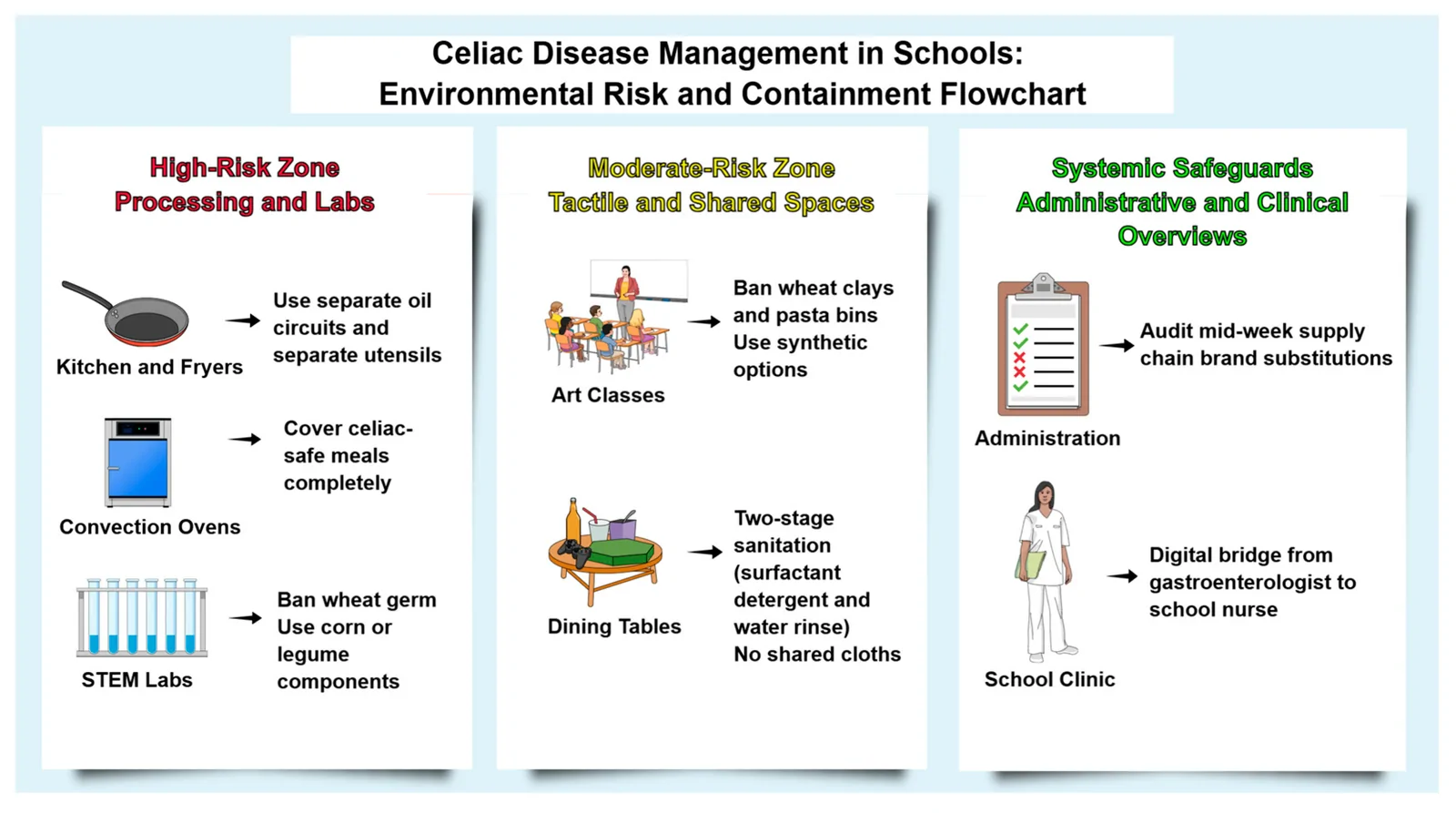
Celiac Disease Management in Schools: Environmental Risk and Containment Flowchart. **Note: This figure is an original work designed and created by the author Alexandra Mpakosi using the Mind the Graph platform (mindthegraph.com).**

To ensure the safe decontamination of environmental surfaces, schools must implement a strict, two-stage sanitation protocol. First, staff must perform a targeted wash using a cleaning agent containing surfactant detergents paired with disposable paper towels to mechanically break down and lift protein residues. Second, the surface must be rinsed with clean water to flush away any remaining traces of the protein matrix from desks or dining tables [33]. Furthermore, the deployment of a single, shared damp cloth or sponge across multiple cafeteria tables is strictly prohibited, as it serves as a primary vector for spreading invisible cross-contact films across the dining environment (Table 4 and Figure 2) [30].

## 9. Limitations of the Study

Several limitations should be considered when interpreting this review. First, as a narrative synthesis, it lacks the formal statistical pooling, meta-analysis, and systematic risk-of-bias assessments characteristic of systematic reviews. Second, the included literature exhibits substantial methodological heterogeneity, blending small qualitative cohorts with quantitative food science tracer experiments.

Furthermore, direct, empirical data on celiac-specific cross-contamination kinetics within actual school environments remains limited. As a result, several operational recommendations are extrapolated from studies in commercial restaurants, hospital catering, food manufacturing plants, and adult nutrition models. Similarly, some psychosocial support strategies were adapted from pediatric food allergy frameworks, which may not completely reflect the strict sub-20 ppm trace-ingestion risks specific to CD autoimmunity.

Finally, the systemic barriers and policy frameworks proposed here are heavily dependent on geographic context. National educational infrastructure, school nurse allocation ratios, public funding, and legal accommodation frameworks vary widely, which may limit the direct scalability of these interventions across different school districts.

## 10. Conclusions

Managing pediatric CD within the school ecosystem requires a holistic approach that considers the intersection of a student’s physical safety, emotional development, and nutritional status. The heterogeneous evidence reviewed suggests that inadequate environmental mitigation measures—such as a reliance on alcohol-based sanitizers or the use of wheat-flour modeling clays—may introduce unintended exposure pathways that compromise a child’s health and academic focus. Furthermore, social containment or protective isolation strategies (for example separate dining tables) may inadvertently foster peer stigmatization and elevate risks for childhood anxiety. Nutritional management should evolve past highly processed, starch-heavy alternatives that can drive metabolic fluctuations, prioritizing instead individualized, dietitian-supervised meal planning centered on nutrient-dense, naturally gluten-free foods. Ultimately, transforming schools into supportive learning environments requires moving away from reactive approaches toward a standardized, multidisciplinary care model. While implementation remains dependent on regional public health resources and legal structures, establishing flexible operational frameworks—including staff education, kitchen risk controls, and equitable meal access—remains essential to supporting both the medical requirements and the social development of students with CD.

## Figures and Tables

**Table 1:** Celiac Disease Risks and Operational Failures in Schools.

School Setting	Risk Type and Vector	Mechanical or Biological Mechanism	Clinical Assessment/Impact
**Cafeteria**	Convection Oven-Air Currents	High-velocity fans circulate loose wheat particles and flour dust across adjacent trays.	Airborne trace deposition potentially breaches the commercial sub-20 ppm safety threshold.
**Cafeteria**	Shared Thermal Frying Oil	Standard frying temperatures do not denature stable gluten; gliadin proteins remain actively suspended in the oil medium.	Introducing measurable quantities of gliadin into naturally gluten-free items.
**Cafeteria**	Shared Service Tools	Mechanical utilization of a single ladle or cloth across multiple food lines transfers an invisible protein film.	Triggers mechanical cross-contact; standard macroscopic checks fail to identify trace molecular residues.
**Classroom**	Tactile Supply Exposure	Wheat-flour modeling clays, raw pasta bins, and papier-mâché coat exposed skin cells.	High frequency of unconscious hand-to-mouth movements poses a risk of accidental non-dietary ingestion.
**STEM Lab**	Powdered Agricultural Media	Biology DNA extractions utilize powdered wheat germ.	Airborne particulate matter colonizes communal workstations, facilitating ingestion via surface contact.
**Restrooms and Halls**	Hygiene and Sanitizer Reliance	Alcohol sanitizers fail chemically to denature inert proteins; they merely dilute and spread gliadin peptides.	Hand sanitizer reliance leaves active residues intact; requires surfactant soap scrubbing and rinsing.
**Social Environment**	Institutionalized Segregation	Assignment to isolated tables and exclusion from group activities labels the child publicly.	May contribute to peer stigmatization, medical bullying, self-exclusion, and heightened anxiety.
**Socio-Economic**	Sourcing and Legal Barriers	Lower-resource public schools lack separate appliances; diagnostic costs delay official medical documentation.	Exacerbate food insecurity as vulnerable children forfeit safe, subsidized school meal programs.

**Table 2:** Systemic Cross-Contamination Risks and Operational Failures in Food Services and Institutional Environments by Country.

Country of Origin	Study Setting and Sample Size	Contamination Risk Detected	Key Critical Control Points (CCPoints) Identified	Source
**Italy (Piedmont Region)**	Institutional catering compliance audits across 277 primary and secondary schools.	71% operational deficit (failed at least one critical safety parameter).	Sourcing tracking, transport contamination, lack of self-control safety plans, and distribution line overlap.	[15]
**United States**	Quantitative simulated school environments (Play-Doh, sensory tables, pasta bins, art supplies). 30 pediatric participants.	Significant molecular transfer following wet matrices (papier-mâché, cooked pasta).	Friction-based hand hygiene with surfactant soap and water is mandatory; dry pasta and raw clay demonstrated minimal transfer when dry.	[33]
**United States**	Commercial restaurant simulation evaluating shared kitchen infrastructure. Multicenter preparation panels.	Inconsistent trace rates varying highly by tool usage.	Shared thermal frying oil, common toaster surfaces, and uncovered convection oven environments.	[30]
**Switzerland**	Experimental household and catering appliance mapping (wooden spoon, colander, serving spoon, and knife). Controlled assays.	Shared ladles pose a higher risk for contamination of gluten-free foods than do shared wooden spoons, colanders, or knives.	Proper mechanical cleaning of kitchen utensils minimizes the health risk associated with gluten.	[27]
**Global Meta-Analysis**	Systematic tracking of commercial food service venues across 17 countries (25,672 food samples).	Meals prepared in food services showed a 4.66% contamination rate, which is lower than raw grains but still presents risk.	Strict adherence to physical cleaning procedures for kitchen equipment and addressing gaps in comprehensive staff training.	[19]
**United Kingdom**	Experimental commercial kitchen trial assessing airborne flour dust transfer during simultaneous prep.	High risk of airborne transfer. A distance of 2 m from the use of wheat flour was required to control gluten contamination at ≤20 ppm if wheat flour had been in use for 4.0 h.	Maintaining a minimum 2 m distance away from the site of wheat flour use, along with good hygiene practices may be effective when preparing gluten-free meals.	[60]
**Brazil**	Evaluation of accidental gluten contamination in 180 traditional lunch meals from food services.	2.8% of analyzed food service dishes presented significant gluten contamination.	Cross-contact during meal plating and serving; handler hygiene and awareness protocols.	[61]

**Table 3:** Whole-Food School Meal Substitution Matrix.

	Avoid (NOVA Group 4: Ultra-Processed Snacks)	Eat Instead (NOVA Group 1: Minimally Processed Whole Foods)	Clinical Context and Dietary Guidance
Breads and Carbohydrate Bases	White rice bread, corn wraps.	Pseudocereals (quinoa, amaranth) or whole grains (wild rice, millet), and sweet potatoes [79].	May support sustained energy levels and help reduce academic cognitive fatigue.
Meats and Protein	Chicken nuggets, packaged deli meats.	Roasted chicken, lean beef, boiled eggs [17,69].	Provide bioavailable iron; may contribute to reducing chronic fatigue secondary to micro-anemias.
Iron Boosters	High-fructose fruit juices, sugary purees.	Bell pepper strips, fresh strawberries [80,81].	Provide an ascorbic acid catalyst; may help optimize non-heme iron absorption across damaged mucosal barriers.
Crunchy Snacks	Commercial potato chips, corn puffs.	Roasted chickpeas, pumpkin seeds [69].	Naturally gluten-free trace minerals; provide zinc and magnesium to support physiological growth requirements.
Healthy Fibers	Packaged fiber bars, peeled fruit cups.	Unpeeled cucumbers [17].	Natural prebiotic fibers may help regulate bowel motility and support bowel regularity.
Milk and Dairy Alternatives	Unmanaged dairy, unfortified alternative milks.	Clinically vetted, fortified plant beverages or low-sugar kefir/Greek yogurt (if dairy is tolerated) [69,82].	Manage secondary lactose intolerance under clinical guidance; support structural calcium density and skeletal mineralization.

**Table 4:** Recommended Interdisciplinary Policy Actions.

Stakeholder Responsible	Core Operational Objective	Action Required	Expected Outcome
**Nurses and Physicians**	Clinical–Educational Integration	Share diagnostic criteria from the physician directly with the school health team.	Ensures that institutional health adjustments are clinically aligned and legally protected.
**School Counselors**	Psychosocial Wellbeing Monitoring	Observe unstructured meal hours; monitor affected students for signs of social withdrawal.	Aids in mitigating peer medical bullying and school isolation.
**School** **Administration**	Staff Education	Deploy annual training defining celiac disease as a chronic autoimmune disorder.	Mitigates misconceptions regarding gluten-free diets across instructional and janitorial teams.
**Cafeteria Managers**	Food Service Risk Containment	Implement separate fryer oil circuits, dedicated utensils, and covered convection trays.	Restricts inadvertent gluten cross-contact during mass production.
**Teachers and Boards**	Classroom Safety Optimization	Exclude wheat-based playdough, raw pasta sensory bins, and wheat-germ laboratory media.	Fosters an inclusive learning environment without risk of non-dietary exposure.
**Procurement Staff**	Supply Chain Accessibility	Procure naturally gluten-free staple grains in bulk; secure funding for safe options.	Alleviates regressive cost burdens for socioeconomically vulnerable families.
**Janitorial Staff**	Environmental Surface Sanitation	Exclude shared damp cloths; clean dining surfaces using surfactant detergents and water.	Mechanically removes stable, sticky gluten protein residues from desks and tables.

## Data Availability

No new data were created or analyzed in this study. Data sharing is not applicable.
